# Prevalence and Predictors of Dietary and Nutritional Supplement Use in the Australian Army: A Cross-Sectional Survey

**DOI:** 10.3390/nu11071462

**Published:** 2019-06-27

**Authors:** Bradley Baker, Bianka Probert, Diane Pomeroy, Julia Carins, Katie Tooley

**Affiliations:** 1 Food and Nutrition, Land Division, Defence Science and Technology Group, Scottsdale, TAS 7260, Australia; 2 Cognition and Behaviour, Land Division, Defence Science and Technology Group, Edinburgh, SA 5111, Australia; 3 Social Marketing @ Griffith, Griffith Business School, Griffith University, Nathan, QLD 4111, Australia

**Keywords:** nutrition, military, human performance, dietary supplements, nutritional supplements

## Abstract

Dietary supplements (DSs) and nutritional supplements (NSs) can enhance performance, recovery or training adaptations, however, some substances, dosages, and usage protocols are unsafe. Knowledge of the type and extent of use within populations enables strategies to be formulated to promote safe and effective use (where needed) and to avoid adverse side effects. The purpose of this study was to understand DS and NS use by active-duty Australian soldiers. Surveys were distributed by e-mail and hard copy to eligible participants (*n* = 23,195). Respondents (males *n* = 1833; females *n* = 296) comprised 9.3% of the total population. Use of ≥1 DSs/week was reported by 76.4% of males and 86.8% of females, and use of ≥1 NSs/week was reported by 21.7% of males and 20.9% of females. The most commonly used supplements were protein or amino acids (55.6%), multivitamins and minerals (38.2%), other DSs (37.8%), individual vitamins and minerals (33.0%), and combination products (32.8%). Logistic regression revealed the number of DSs respondents used simultaneously was significantly different between males and females, age groups, BMI ranges, and body weight actions. Engagement in special operations was a significant predictor of the use of any DS, individual vitamin and minerals and multivitamin and minerals. Approximately 16% of regular DS users reported experiencing one or more side effects, with the most common being palpitations (10.6%), tingling or numbness in the face, fingers, arms, or legs (5.5%), tremors or shaking (2.9%), flushing (2.3%), headache (2.0%), abdominal pain (1.6%), anxiety (1.4%), and dizziness or confusion (0.9%). The results revealed more prevalent use of several categories of DSs and NSs among some subgroups. Ongoing surveillance of DS and NS use is important for tracking trends in use over time and gauging the effectiveness of any strategies employed to enhance the quality of supplement use.

## 1. Introduction

Dietary supplements have been described as “a food, food component, nutrient, or non-food compound that is purposefully ingested in addition to the habitually-consumed diet with the aim of achieving a specific health and/or performance benefit” [1]. From this broad definition, two sub-groups can be formed as follows. Dietary supplements (DSs) include multi-vitamins and minerals, individual vitamins and minerals, protein and amino acids, purported prohormones, herbal (plant-derived) substances, joint health products, combination products, and other DSs (plant, animal and synthetic substances) [2,3]. Nutritional supplements (NSs) include products formulated for use before, during and after exercise, such as sports drinks, energy bars and gels, and meal-replacement beverages [3].

Several DSs and NSs are supported by “good to strong” evidence of enhancing aspects of performance, recovery, or training adaptations when used correctly [1]. However, many others are unsafe, with some linked to dangerous side effects [4,5]. In addition, some supplement dosages and protocols can increase the risk of dangerous side effects, such as the simultaneous use of multiple products [6]. These risks are acknowledged by militaries internationally in policies and education on the use of dietary supplements [7,8]. The proportion of the Australian population regularly using DSs is approximately 35% of men and 50% of women [9,10]. However, the prevalence of both DS and NS use in non-Australian military populations has been found to exceed levels in general populations [11].

Active-duty soldiers show particular interest in the effectiveness of DSs and NSs in enhancing aspects of their job performance, fitness, recovery, and health (such as bone health and gut health). A recent study investigated the use of 19 generic DSs among physical training instructors, cooks, and headquarter-based personnel in the Australian Army, and found use of one or more DSs was common [12,13]. However, the prevalence and extent of use of a wider range of supplements, as well as supplement use among other occupations, may vary. The purpose of the present study was to investigate the use of a comprehensive range of DSs and NSs by active-duty Australian soldiers across a range of occupations, and to further our understanding of supplement use associations with demographic and occupational characteristics, weight goals, and physical activity levels. This knowledge will enable education and other strategies to be formulated to promote safe and effective use (where needed) and to avoid adverse side effects.

## 2. Materials and Methods

### 2.1. Sampling and Recruitment

This study was approved by the Australian Defence Human Research Ethics Committee (now the Departments of Defence and Veterans’ Affairs Human Research Ethics committee) and given the protocol number 847-16. Data collection occurred between February and September 2017. Active-duty Australian Army soldiers aged 18 years and over were emailed the study description and online survey link by e-mail with an invitation to participate in the study. Potential participants were informed that the survey would remain open for four weeks, and a follow-up email was sent after two weeks. Paper surveys were distributed at six Australian Army regiments. These were chosen to capture soldiers with differing ranks and occupations who do not primarily undertake desk-based duties or regularly access Army computer terminals (which would be required for completing the online survey)—the Special Air Service Regiment, the 2nd Commando Regiment, the 1st Battalion, Royal Australian Regiment, the 3rd Battalion, Royal Australian Regiment, the 2nd Cavalry Regiment, and the 3rd Combat Engineer Regiment. All written and verbal study information stated that participation was voluntary and anonymous.

### 2.2. Survey

The survey investigated the use of any DS and NS in the past six months and was constructed in part from questions sourced from Lieberman et al. [2]. The survey was then amended to reflect the contemporary DS/NS market and ensure questions asked were appropriate for an Australian military audience. The survey was then piloted and further refined among six Australian Army officers and four military human sciences researchers who were asked to provide feedback throughout its development relating to suitability and face validity. Demographic and military characteristics questions included gender, age group, height, body weight, Corps, command, and rank. Questions on physical activity and body weight included frequency and duration of cardiorespiratory exercise undertaken per week, number of strength training sessions undertaken per week, and body weight goals. A total of 70 generic DSs and NSs were listed across eight DS and three NS categories as described by Lieberman et al. [2] and Austin et al. [2,11]. Respondents were asked if they used any of the listed products or any similar product/s. If a respondent answered “yes”, they were presented with a table listing each supplement and six “other” spaces and were asked to indicate the frequency of their use of each on a scale (i.e., never, once a month, once a week, 2–6 times per week, or daily).

### 2.3. Statistical Analysis

Statistical analyses were performed using the Statistical Package for the Social Sciences (SPSS) version 24 (IBM Corp, Armonk, NY, USA) [14]. The percentage of participants (± standard error) who reported regular use of DSs and NSs (i.e., use ≥1 times/week) was calculated to determine the prevalence of regular use. Prevalence of side effects of DS use was calculated as the percentage (± standard error) of regular DS users who reported side effects. The mean (± standard deviation) number of DS and NS products used was calculated. Body Mass Index (BMI) in kg/m^2^ was calculated from participants’ reported body weights and heights. Weekly duration of cardiorespiratory exercise was calculated from reported frequency and duration of sessions. If a respondent indicated their frequency of cardiorespiratory sessions was multiple daily sessions throughout each week, it was estimated they undertook 10 sessions per week. Logistic regression analysed associations between use of each category of DS and NS and explanatory variables including demographic and military characteristics, and number of strength training sessions per week. Negative binomial regression analysed associations between the mean number of DSs and NSs used, and the explanatory variables (i.e., demographic characteristics, military characteristics, and body weight actions) and continuous variables (i.e., BMI, hours of cardio exercise undertaken per week, and number of strength training sessions undertaken per week). The level of statistical significance was set at *p* < 0.05.

## 3. Results

A total of 22,743 soldiers received an e-mail invitation to the survey, and 1777 (7.8%) of these completed it on-line. Of the 452 potential participants who were verbally briefed and provided with hard copies of the participant information and survey, 385 (85.2%) returned completed surveys. One response was excluded due to the report of an implausible number of DSs and NSs—70 products per day. A total of 2162 respondents (1833 males and 296 females) was included in the final data set, giving an overall response rate of 9.3%.

### 3.1. Categories of DSs and NSs Used

Overall, regular use (i.e., ≥1 times/week) of a total of 148 types of DSs and eight types of NSs was reported by respondents. The regular use of any DS and/or NS was reported by 78.7% and 88.9% of males and females, respectively. Females were significantly more likely to be regular users of DSs than males (*p* < 0.001), with 76.4% of male and 86.8% of female soldiers using DSs ≥ 1 times/week (see Table 1 for DS use prevalence and Table 2 for odds ratios (ORs)).

NSs were used to a lesser extent than DSs, with 21.7% and 20.9% of males and females using NSs ≥ 1 times/week, respectively (see Table 3). ORs for NS use are shown in Table 4.

Compared to males, females were significantly more likely to use any DS, multivitamin and minerals, individual vitamins or minerals, and herbal products. Males appeared more likely to use purported prohormones compared to females, however this did not reach statistical significance (*p* = 0.075). Gender was not a significant predictor of use for any other categories of DSs and NSs. Age group was a significant predictor of the prevalence of use of any DS. Soldiers aged 23–27 were more likely to be NS users compared to their younger counterparts, with age group significantly predicting the use of any NS. Across the other DS and NS categories, age group also significantly predicted the use of multivitamin and minerals, protein or amino acids, combination products, herbal products, other DSs, and sports drinks. Soldiers belonging to Special Operations Command (SOCOMD) were significantly more likely to use any DS, multivitamin and minerals, and individual vitamin and minerals than soldiers belonging to other commands. BMI as a continuous variable predicted the use of purported prohormone products, joint health products and other DSs, with increases of 9%, 6% and 4% in the likelihood of use per additional kg/m^2^, respectively. Soldiers in higher BMI ranges—those with a BMI of 25–30 kg/m^2^ (*p* = 0.001) and >30 kg/m^2^ (*p* = 0.022) were significantly more likely to use combination products compared to those with a BMI < 25 kg/m^2^. Respondents trying to gain weight were more likely to use protein or amino acids and combination product supplements than those reporting other body weight actions. The odds of the use of all categories of DSs, except joint health products, increased with higher levels of use per additional strength training session. Increases in hours of cardio exercise/week were a significant predictor of the use of any DS (i.e., from any category), combination products, protein or amino acids, individual vitamins and minerals, other DSs, sports drinks, meal replacement beverages, and any NS. Specifically, higher hours of cardio exercise/week within respondents’ own time and within their unit were predictors of the use of any DS, combination products, and protein or amino acids. Additionally, there was a 5% increase in the likelihood of individual vitamin or mineral use for each additional hour of cardio exercise undertaken in respondents’ own time. The use of other DSs increased by 3% per additional hour of cardio exercise undertaken per week in total.

### 3.2. Side Effects of DS Use

The prevalence (% ± SE) of regular DS users who reported one or more side effects was 15.9 ± 0.1 (*n* = 267). The number of DS products regularly used by those who reported one or more side effects was 7.6 ± 6.1, compared to 4.7 ± 4.2 among those who did not report side effects. The most commonly reported side effect of regular DS use was palpitations (10.6 ± 0.01) followed by tingling or numbness in the face, fingers, arms, or legs (5.5 ± 0.01), tremors or shaking (2.9 ± 0.00), flushing (2.3 ± 0.00), headache (2.0 ± 0.00), abdominal pain (1.6 ± 0.00), anxiety (1.4 ± 0.00), dizziness or confusion (0.9 ± 0.00), low mood (0.8 ± 0.00), and other not disclosed (1.0 ± 0.00). A total of 0.1% ± 0.01% of regular DS users reported side effects including mood changes (e.g., mood swings), pimples, and/or constipation. DS users who reported anxiety used the highest number of DSs regularly, 11.5 ± 8.0 products. The number (mean ± SD) of strength training sessions undertaken by regular DS users who reported one or more side effects was 5.8 ± 2.9, which was greater than that undertaken by all survey participants (4.4 ± 2.8). Table 5 details the characteristics of regular DS users who reported one or more side effects.

### 3.3. Types of DSs and NSs Used

Overall, the most popular type of protein and amino acid DSs used ≥1 times/week were protein powders, which were used by 49.4% of all respondents (*n* = 1069). These were followed by branched chain amino acid mixtures and other amino acid mixtures (*n* = 654; 30.3%), creatine (*n* = 342; 15.8%), protein bars (*n* = 255; 11.8%), glutamine/L-glutamine (*n* = 170; 7.9%), carnitine/L-carnitine (*n* = 139; 6.4%), beta-alanine (*n* = 123; 5.7%), and various other types were used by 19.1% of respondents (*n* = 413). Multivitamin and mineral DSs and other DSs were the second and third most commonly used DS categories—the most commonly used types of the latter were long-chain omega-3 (*n* = 673; 31.1%), probiotics (*n* = 194; 9.0%), fibre supplements (*n* = 123; 5.7%), coenzyme Q10 (*n* = 38; 1.8%), beetroot juice (*n* = 25; 1.2%), melatonin (*n* = 21; 1.0%), and a variety of others (*n* = 63; 2.9%). The most popular types of combination products were “pre-workout”/“intra-workout” supplements (*n* = 602; 27.8%), “fat burner”/“thermogenic” supplements (*n* = 252; 11.7%), and “post-workout” supplements (*n* = 234; 10.8%). A further 6.5% of respondents (*n* = 141) used various other types of combination products. Individual vitamins and minerals were the fourth most commonly used category of DS, with the most commonly used types being magnesium (n = 392; 18.1%), vitamin C (*n* = 288; 13.3%), zinc (*n* = 209; 9.7%), vitamin D (*n* = 126; 5.8%), vitamin B12 (*n* = 105; 4.9%), iron (*n* = 91; 4.2%), calcium (*n* = 77; 3.6%), vitamin E (*n* = 34; 1.6%), and others (*n* = 91; 4.2%). The most popular herbal products were garlic (*n* = 124; 5.7%), ginger (*n* = 94; 4.4%), echinacea (*n* = 46; 2.1%), ginseng (*n* = 39; 1.8%), gingko (*n* = 33; 1.5%), caffeine tablets/gum (*n* = 31; 1.4%), turmeric/curcumin (*n* = 22; 1.0%), followed by various others (*n* = 85; 3.9%). Glucosamine (with or without chondroitin) was the most commonly used joint health product, used by 9.6% of respondents (*n* = 207). The most commonly used type of purported prohormones were those which purported to boost testosterone levels—used by 4.7% of respondents (*n* = 102), followed by various others (*n* = 54; 2.5%). As shown in Table 4, sports drinks were the most commonly used NS—regular use was reported by 19.5% (*n* = 422) of respondents. These were followed by sports bars and gels, which were used by 4.5% (*n* = 97) of respondents.

### 3.4. Number of DSs and NSs Used

All respondents used 4.0 ± 4.6 (mean ± SD) DSs/week, with higher numbers used by females (4.7 ± 4.8) compared to males (3.9 ± 4.6). Higher numbers of DSs were used by soldiers aged 23–27 (5.2 ± 5.4), 28–32 (4.8 ± 4.7), and 33–37 (4.6 ± 4.9), compared to soldiers in other age groups (58–62 (2.4 ± 2.6]), 53–57 (2.5 ± 3.0), and 43–47 (2.6 ± 3.6)). Table 6 displays the *p* values, ORs and 95% CIs for significant predictors of the number of DSs used/week. Pearson’s correlation coefficients are shown in Table 7 for all continuous variables. There was a moderate positive correlation between number of strength training sessions per week and total number of DSs used, and a weak positive correlation between hours of cardio exercise undertaken per week and total number of DSs used, as shown in Table 7.

All respondents used 0.28 ± 0.60 NSs/week, with age group a significant predictor of the number used. Those aged between 23 and 27 reported using the highest number—0.37 ± 0.66 NSs/week. Hours of cardio exercise in own time/week was weakly correlated with number of NSs used/week, with a 0.06% increase in the number of supplements used per additional hour.

## 4. Discussion

Knowledge of the types of DSs and NSs used within populations enables strategies to be formulated to promote safe and effective use (where needed) and to avoid adverse side effects. Accordingly, this study investigated active-duty Australian soldiers’ use of a comprehensive range of DSs and NSs. Consistent with other studies in military and civilian populations, this study found the prevalence of supplement use was higher in females than males [9,10,15]. However, this contrasts with another recent study of Australian soldiers’ supplement use (published whilst data collection was underway) which found lower use by females than males (70.0% and 83.2%, respectively) [12]. The differing findings between the present study and those reported by Kullen et al. [12] may be due to the inclusion of a larger range of DSs and NSs in the present study—particularly in the categories of vitamins and minerals, combination products, herbal products, and other DSs, as females tended to use higher levels of these supplements. Higher use of vitamin and mineral supplements—iron and vitamin C in particular—by females has previously been observed in Australia [16]. Highly active female soldiers may indeed require supplementation more often than males, considering studies have shown females’ iron levels decline during periods of heavy Army training, which adversely affects fatigue levels, mood, and physical performance [17,18]. Health professionals may recommend vitamin and mineral supplements to females for other reasons, such as calcium for osteoporosis prevention and folate for foetal development [19,20]. Across the other categories of DSs, females were slightly more likely to use combination products and herbal DSs than males, and the extent of simultaneous use of multiple DSs was greater in female soldiers.

DS use by this military population was greater than that reported in the general Australian population—76% of males and 86% of females compared to 35% of males and 50% of females in the general population [9,10]. A study of DS use in a predominantly female (76%) sample of an Australian university population found that overall, 74% of respondents used DSs in the previous six months [16]. In the study reported herein, males comprised ~85% of all respondents, and remembering we investigated weekly DS use, we found ~77% of respondents used DSs. Noting these differences, compared to the Australian university population, we report a substantially higher prevalence of use of protein powders (~50% vs. ~30%) and amino acids (~30% vs. ~7%) in particular. In addition, ~33% of this military population used combination products, with ~28% using “pre-workout”/“intra-workout” products, and the level of use of these products was not reported on in the Australian university population [16]. Military personnel have reported higher overall DS usage than the civilian population in the U.S. as well [11,21,22]. DS use observed in this study was greater than that of U.S. Army personnel (~63% of male and ~71% of female soldiers regularly used DSs; Austin et al. [11]), and was also greater than that of British soldiers (38% were current users of DSs; Casey et al., [23]). However, time has passed since these studies were undertaken in the U.S. and Britain, and the higher usage in this study may be partially explained by the rising trends of use observed over time in both military and general populations [11].

Across demographic and military characteristics, the highest numbers of DSs were used by soldiers aged between 23 and 37, those belonging to SOCOMD (i.e., Special Forces), those with ranks between trainee and corporal, as well as those with ranks between officer cadet and junior commissioned officer. Additionally, soldiers with goals related to body weight, i.e., trying to gain weight; soldiers trying to maintain weight; as well as those in higher BMI ranges; also used higher numbers of DSs. Such groups may be more likely to seek enhanced muscle adaptations through use of DSs such as protein and amino acids [1,3]. Conversely, soldiers aged 43 and over were less likely to use protein or amino acid DSs than their younger counterparts. This may be due to the different occupational characteristics of soldiers in older vs. younger age groups and/or a lower frequency of strength training sessions in older vs. younger age groups. Across all categories of DSs, all except joint health products were more frequently used by soldiers who undertook higher levels of strength training. Soldiers undertaking high levels of physical activity and athletes alike may require other supplementation to meet requirements or treat deficiencies—in particular iron, calcium, and vitamin D [1,17,24,25]. Self-administration of DSs carries heightened risks of side effects, and any recommendation by a dietitian or doctor to use a DS should be accompanied by education on safe and effective use. Monitoring is indicated for certain dosages of some DSs to avoid toxicity (e.g., vitamin D) or other adverse effects (e.g., beta-alanine) [1,26,27].

The overall proportion of respondents who regularly used combination products (~33%) is higher than the proportion found in the U.S. Army (~24%) [11]. DSs sold both internationally and in Australia—combination and purported prohormone products in particular—have been found to contain both World Anti-Doping Agency (WADA) prohibited substances and border-controlled substances [28,29,30]. Recent detections of border-controlled substances in DSs sold in Australia have included dangerous stimulants (e.g., dimethylbutylamine or “DMBA”) and anabolic androgenic steroids [29,30]. Furthermore, some purported prohormones may evade detection in batch testing, as they are not detectable as androgens until they are metabolised in the body [30]. These issues increase the risk that DS use could have consequences for the careers of Australian Defence Force (ADF) members, as use of WADA prohibited substances contravenes ADF policy [7]. Members who test positive for border-controlled substances in the ADF’s Prohibited Substance Testing Program may be discharged from service. This is highlighted by a number of recent reports of dismissed Army members due to testing positive for steroids [31,32,33,34].

The use of combination products, which may contain between 10 and 30 DS ingredients, and/or multiple performance-related DSs carries an increased risk of side effects and may indeed be counterproductive to performance [3,6]. Due to the heightened risk of side effects from combining DSs, the use of multiple performance-related DSs simultaneously is not recommended [6]. In this study, common self-reported side effects of regular DS use included palpitations, tingling or numbness in the face, fingers, arms, or legs, tremors or shaking, headache, abdominal pain, anxiety, and dizziness or confusion. While many types of DSs may cause side effects, combination products for energy (e.g., “pre-workout” and “nitric oxide supplements”) and weight loss (e.g., “fat burners”), were among the most popular types of supplements used by respondents. A study of DS use in U.S. Navy and Marine Corps personnel found that combination products resulted in the largest proportion of adverse events [3]. An analysis of U.S. hospital emergency department presentations found that combination products for weight loss and energy resulted in adverse events, with symptoms including the side effects reported by participants in our study as well as rapid heartbeat, loss of consciousness, and blurred vision [5]. In Australia, symptoms of psychosis including psychomotor retardation, thought broadcasting, auditory hallucinations, and persecutory delusions were recently attributed to the use of a combination product for weight loss [35]. Also recently, weight loss DSs containing 2,4-dinitrophenol caused several deaths both in Australia and overseas [36]. Another concern for the study population is the impact that some side effects could have on their military performance and effectiveness.

NSs were used to a lesser extent than DSs, and most commonly by respondents in the mid-20s age group. The levels of NS use in this study were considerably lower than have been found in other military populations, e.g., British soldiers [23]. In the present study, ~19% of respondents regularly used sports drinks; comparatively ~50% of British soldiers were found to be current users of sports drinks [23]. Also in the present study, ~10% of the participants reported following the low-carbohydrate, high-fat diet, and anecdotally the practice of such diets is increasing. Also anecdotally, there is increasing avoidance of sports drinks among Australian soldiers due to their sugar content. Indeed, limiting dietary intake of added sugars is an increasing health concern in the western world, and these trends may explain the lower use of sports drinks found in the present study. However, research shows that, in addition to aiding hydration, sports drinks are beneficial to endurance exercise capacity during prolonged and/or intermittent exercise. When consumed in appropriate quantities and according to individual tolerance, they provide the body with a source of carbohydrate to use as fuel when glycogen stores are stressed [37].

Population level strategies to support safe and effective DS use and reduce potentially dangerous DS use should be developed and evaluated. However, little exists to guide the development of such strategies. In the sporting arena, behavioural change strategies that have involved educative strategies (particularly information provision and awareness raising in isolation) have resulted in poor outcomes—increasing knowledge, but having no clear impact on attitudes or intentions to use unsafe, banned DSs [38]. Meta-analyses have found attitudes, perceived norms, and self-efficacy (to refrain from use) were significant predictors of doping intentions, and as a result, interventions should attempt to de-normalise use in sports and exercise settings [39]. Behavioural change strategies with components designed to develop skills, change social norms or encourage goal setting are considered more promising than information provision [38]. Development of strategies for a military population will be reliant on understanding the motivators for supplement use; the benefits and barriers associated with those behaviours, as well as those associated with new or alternative behaviours. Strategies developed with this understanding are more likely to resonate with personnel; to change soldiers’ attitudes and values towards unsafe DSs; and to motivate a change in their intentions to use them [40].

In the dynamic evolving DS and NS market, new products, substances, and analogues often replace those which have been found to be unsafe and/or ineffective. In Australia, this has led to loopholes in the regulation of DSs and NSs—allowing potentially unsafe products to enter the market [41]. Closing these loopholes, as well as increasing the enforcement capacity of Australia’s regulators—including enabling enforcement officers to more efficiently remove non-compliant products from the market—were options that were recently suggested during a roundtable discussion attended by representatives from Australian Government agencies, Australian state and territory governments, public health organisations, and from the supplement industry [41]. These measures would serve to improve the safety of DS use, by reducing the local availability of products that are adulterated with unsafe and/or undeclared substances.

Ongoing surveillance of DS and NS use should continue to identify current and past trends in use. Future studies should monitor these patterns of use over time within the Army context and should extend to examine whether patterns of use are similar within the Australian Air Force and Navy, given that their roles differ from Army. This is particularly important due to the evolving nature of the DS and NS market, with new and reformulated products appearing regularly, and the trend for increased use that has been observed in other military forces [11]. Surveillance will enable the monitoring and evaluation of the effectiveness of strategies employed to alter patterns of DS use, which should focus on the prevention/reduction of potentially unsafe DS use and the promotion of evidence-based, performance-enhancing DSs and NSs.

## 5. Limitations

A large number of soldiers responded to the survey; however the overall response rate from all soldiers contacted was low (9.3%). The response rate from those provided with hard-copy surveys was considerably higher compared to that of those who received an e-mail notification of the study. As a result, there are limitations in how generalizable the findings of this study are to the entirety of the Australian Army. Another limitation of this study is that self-reported information may be biased (e.g., by recall bias and social desirability bias). Finally, factors such as caffeine intake, pre-existing conditions, and medications may increase the risk of side effects from DS use and/or cause some of the side effects reported by participants in this study. Consequently, there is a degree of uncertainty that the side effects reported by participants in this study can be attributed solely to DS use.

## 6. Summary

This study demonstrates that DS and NS use in the Australian Army is more widespread than in the general Australian population, and a considerable proportion of DS users reported one or more adverse side effects. The findings of this study can be used as a basis for behavioural change strategies designed for consumers and improved governance to modify the availability of DSs and NSs. Behaviour change strategies should move beyond information provision and education to attitudinal change and de-normalising use of potentially harmful supplements, with research continuing to focus on determining the safety and effectiveness of supplements that soldiers are most likely to use. Strategies should focus on promoting the use of quality DSs and NSs (those that are independently tested and evidence-based) avoiding adverse side effects. Ongoing surveillance of DS and NS use is important for tracking trends in use over time and gauging the effectiveness of strategies employed to alter patterns of DS use.

## Figures and Tables

**Table 1:** Prevalence and predictors of the use of each category of dietary supplement (DS) ≥ 1 times/week by demographic, military, and lifestyle characteristics ^1^.

Variable	Subgroup	Any DS (% ± SE)	Multivitamin and Mineral (% ± SE)	Individual Vitamin or Mineral (% ± SE)	Protein or Amino Acid (% ± SE)	Combination Product (% ± SE)	Herbal Product (% ± SE)	Purported Prohormone (% ± SE)	Joint Health Product (% ± SE)	Other (% ± SE)
**Group**	All (*n* = 2162)	77.1 ± 0.9	38.2 ± 1.0	33.0 ± 1.0	55.6 ± 1.1	32.8 ± 1.0	13.6 ± 0.7	4.7 ± 0.5	9.6 ± 0.6	37.8 ± 1.0
**Gender**	Male (*n* = 1833)	76.4 ± 1.0	37.6 ± 1.1	30.9 ± 1.1	56.5 ± 1.2	32.4 ± 1.1	12.7 ± 0.8	5.1 ± 0.5	9.3 ± 0.7	37.1 ± 1.1
	Female (*n* = 296)	86.8 ± 2.0 ^##^	43.6 ± 2.9 **	47.3 ± 2.9 ^##^	52.7 ± 2.9	36.5 ± 2.8	18.6 ± 2.3 ^#^	2.7 ± 0.9	10.8 ± 1.8	42.2 ± 2.9
	Prefer not to say (*n* = 18)	61.1 ± 11.5	27.8 ± 10.6	27.8 ± 10.6	33.3 ± 11.1	22.2 ± 9.8	16.7 ± 8.8	0.0 ± 0.0	27.8 ± 10.6	38.9 ± 11.5
	Indeterminate/Intersex/Unspecified (*n* = 11)	63.6 ± 14.5	18.2 ± 11.6	27.3 ± 13.4	27.3 ± 13.4	9.1 ± 8.7	18.2 ± 11.6	0.0 ± 0.0	0.0 ± 0.0	36.4 ± 14.5
	*p* value *	*p* = 0.003 *	*p* = 0.025 *	*p* < 0.001 *	*p* = 0.084	*p* = 0.228	*p* = 0.081	*p* = 0.529	*p* = 0.160	*p* = 0.565
**Age**	18–22 (*n* = 210)	76.2 ± 2.9	24.8 ± 3.0	27.1 ± 3.1	66.2 ± 3.3	31.9 ± 3.2	11.0 ± 2.2	2.9 ± 1.1	1.0 ± 0.7	27.5 ± 3.0
	23–27 (*n* = 457)	85.6 ± 1.6 **	43.3 ± 2.3 ^##^	37.0 ± 2.3	75.5 ± 2.0 **	45.7 ± 2.3 ^#^	12.9 ± 1.6	5.9 ± 1.1	5.9 ± 1.1 **	38.9 ± 2.3 ^#^
	28–32 (*n* = 396)	82.3 ± 1.9	40.9 ± 2.5 ^##^	35.9 ± 2.4	66.7 ± 2.4	43.2 ± 2.5 ^#^	13.1 ± 1.7	5.1 ± 1.1	7.1 ± 1.3 ^#^	37.4 ± 2.4 ^#^
	33–37 (*n* = 293)	81.2 ± 2.3	43.0 ± 2.9 ^##^	37.2 ± 2.8	63.1 ± 2.8 ^#^	37.2 ± 2.8 ^#^	13.7 ± 2.0	5.5 ± 1.3	9.2 ± 1.7 ^#^	45.1 ± 2.9 ^##^
	38–42 (*n* = 225)	76.0 ± 2.8	43.1 ± 3.3 ^##^	33.3 ± 3.1	44.4 ± 3.3	28.9 ± 3.0	18.2 ± 2.6	6.7 ± 1.7	12.0 ± 2.2 ^##^	35.6 ± 3.2 ^#^
	43–47 (*n* = 220)	62.7 ± 3.3	33.2 ± 3.2 ^#^	24.5 ± 2.9	31.8 ± 3.1 **	16.8 ± 2.5	11.4 ± 2.1	3.6 ± 1.3	13.6 ± 2.3 ^##^	35.0 ± 3.2 ^##^
	48–52 (*n* = 188)	66.5 ± 3.4	34.6 ± 3.5 ^#^	31.9 ± 3.4	26.6 ± 3.2 ^##^	13.8 ± 2.5 **	18.1 ± 2.8	3.2 ± 1.3	16.5 ± 2.7 ^##^	38.3 ± 3.5 ^##^
	53–57 (*n* = 125)	76.8 ± 3.8	29.6 ± 4.1	27.2 ± 4.0	32.0 ± 4.2 **	16.8 ± 3.3	10.4 ± 2.7	0.8 ± 0.8	18.4 ± 3.5 ^##^	43.2 ± 4.4 ^##^
	58–60 (*n* = 34)	76.5 ± 7.3	38.2 ± 8.3 **	35.3 ± 8.2	17.6 ± 6.5 ^#^	5.9 ± 4.0 **	11.8 ± 5.5	8.8 ± 4.9	29.4 ± 7.8 ^##^	50.0 ± 8.6 ^##^
	Prefer not to say (*n* = 14)	57.1 ± 13.2	21.4 ± 11.0	14.3 ± 9.4	28.6 ± 12.1	14.3 ± 9.4	14.3 ± 9.4	0.0 ± 0.0	21.4 ± 11.0 ^##^	35.7 ± 12.8
	*p* value *	*p* = 0.003 *	*p* < 0.001 *	*p* = 0.073	*p* < 0.001 *	*p* < 0.001 *	*p* = 0.287	*p* = 0.289	*p* < 0.001 *	*p* < 0.001 *
**Command**	Other than SOCOMD (*n* = 1960)	76.7 ± 1.0	37.1 ± 1.1	31.9 ± 1.1	54.5 ± 1.1	32.7 ± 3.3	13.2 ± 0.8	4.5 ± 0.5	9.7 ± 0.7	37.3 ± 1.1
	SOCOMD (*n* = 202)	86.6 ± 2.4	48.5 ± 3.5	43.6 ± 3.5 ^#^	66.3 ± 3.3	33.7 ± 1.1	17.3 ± 2.7	6.9 ± 1.8	8.9 ± 2.0	42.6 ± 3.5
	*p* value *	*p* = 0.003 *	*p* = 0.011	*p* = 0.001 *	*p* = 0.057	*p* = 0.782	*p* = 0.101	*p* = 0.122	*p* = 0.719	*p* = 0.141
**Rank**	Trainee (*n* = 38)	86.8 ± 5.5	31.6 ± 7.5	34.2 ± 7.7	73.7 ± 7.1	44.7 ± 8.1	10.5 ± 5.0	2.6 ± 2.6	5.3 ± 3.6	42.1 ± 8.0
	Private–Corporal (*n* = 1025)	79.8 ± 1.3	37.3 ± 1.5	35.5 ± 1.5	66.0 ± 1.5	39.9 ± 1.5	13.7 ± 1.1	6.4 ± 0.8	6.4 ± 0.8	37.2 ± 1.5
	Senior NCO (*n* = 217)	79.7 ± 2.7	39.2 ± 3.3	35.0 ± 3.2	54.8 ± 3.4	33.6 ± 3.2	16.6 ± 2.5	5.1 ± 1.5	12.4 ± 2.2	45.2 ± 3.4
	Warrant Officer (*n* = 282)	71.6 ± 2.7	35.8 ± 2.9	31.6 ± 2.8	36.5 ± 2.9	19.1 ± 2.3	12.8 ± 2.0	4.3 ± 1.2	14.9 ± 2.1	35.5 ± 2.8
	Officer Cadet–Junior Commissioned Officer (*n* = 233)	86.3 ± 2.3	44.6 ± 3.3	33.9 ± 3.1	69.1 ± 3.0	40.8 ± 3.2	13.7 ± 2.3	2.1 ± 0.9	9.0 ± 1.9	38.2 ± 3.2
	Senior Commissioned Officer (*n* = 343)	68.2 ± 2.5	39.1 ± 2.6	25.3 ± 2.4	31.5 ± 2.5	16.2 ± 2.0	12.1 ± 1.8	2.1 ± 0.8	12.9 ± 1.8	35.3 ± 2.6
	Prefer not to say (*n* = 24)	70.8 ± 9.3	29.2 ± 9.3	29.2 ± 9.3	33.3 ± 9.6	25.0 ± 8.8	16.7 ± 7.6	0.0 ± 0.0	20.8 ± 8.3	50.0 ± 10.2
	*p* value *	*p* = 0.438	*p* = 0.354	*p* = 0.076	*p* = 0.219	*p* = 0.501	*p* = 0.885	*p* = 0.251	*p* = 0.946	*p* = 0.288
**Corps Area**	Combat (*n* = 913)	74.0 ± 1.5	36.7 ± 1.6	30.4 ± 1.5	56.8 ± 1.6	30.3 ± 1.5	11.3 ± 1.0	4.4 ± 0.7	8.3 ± 0.9	35.6 ± 1.6
	Combat Support (*n* = 261)	83.1 ± 2.3 ^#^	43.7 ± 3.1	36.4 ± 3.0	59.0 ± 3.0	33.7 ± 2.9	16.9 ± 2.3	6.5 ± 1.5	10.0 ± 1.9	44.1 ± 3.1
	Combat Service Support (*n* = 980)	79.5 ± 1.3	38.2 ± 1.6	34.3 ± 1.5	53.6 ± 1.6	34.8 ± 1.5	14.7 ± 1.1	4.6 ± 0.7	10.8 ± 1.0	38.3 ± 1.6
	Officer Training Corps (*n* = 8)	87.5 ± 11.7	37.5 ± 17.1	62.5 ± 17.1	62.5 ± 17.1	37.5 ± 17.1	25.0 ± 15.3	0.0 ± 0.0	0.0 ± 0.0	25.0 ± 15.3
	*p* value *	*p* = 0.024 *	*p* = 0.526	*p* = 0.056	*p* = 0.310	*p* = 0.216	*p* = 0.159	*p* = 0.551	*p* = 0.337	*p* = 0.077
**BMI Range**	<25 (*n* = 701)	76.2 ± 1.6	34.5 ± 1.8	34.0 ± 1.8	55.6 ± 1.9	28.0 ± 1.7	13.4 ± 1.3	3.1 ± 0.7	6.3 ± 0.9	33.7 ± 1.8
	25–30 (*n* = 1114)	79.5 ± 1.2	40.8 ± 1.5	33.3 ± 1.4	58.2 ± 1.5	35.5 ± 1.4 **	13.7 ± 1.0	5.6 ± 0.7	11.8 ± 1.0 ^#^	39.9 ± 1.5
	>30 (*n* = 292)	76.0 ± 2.5	37.3 ± 1.1	30.8 ± 2.7	50.0 ± 2.9	35.5 ± 2.8 **	13.7 ± 2.0	5.1 ± 1.3	9.2 ± 1.7	39.4 ± 2.9
	*p* value *	*p* = 0.562	*p* = 0.168	*p* = 0.629	*p* = 0.278	*p* = 0.028	*p* = 0.980	*p* = 0.006 *	*p* = 0.003 *	*p* = 0.449
**BMI (kg/m^2^) ^†^**	-	-	-	-	-	-	-	-	-	-
	*p* value	*p* = 0.187	*p* = 0.121	*p* = 0.212	*p* = 0.522	*p* < 0.001 *	*p* = 0.335	*p* = 0.006 *	*p* = 0.004 *	*p* = 0.007 *
**Body Weight Actions**	Trying to lose weight (*n* = 810)	78.8 ± 1.4	39.5 ± 1.7	31.1 ± 1.6	51.2 ± 1.8	35.4 ± 1.7	14.2 ± 1.2	4.1 ± 0.7	9.9 ± 1.0	38.8 ± 1.7
	Trying to gain weight (*n* = 276)	89.1 ± 1.9	40.6 ± 3.0	34.8 ± 2.9	84.8 ± 2.2 ^##^	43.5 ± 3.0	13.0 ± 2.0	7.2 ± 1.6	6.5 ± 1.5	35.5 ± 2.9
	Trying to maintain weight (*n* = 544)	82.5 ± 1.6	43.4 ± 2.1	40.4 ± 2.1 ^#^	64.0 ± 2.1	38.1 ± 2.1	15.3 ± 1.5	7.7 ± 1.1 ^#^	11.9 ± 1.4	43.9 ± 2.1
	Not trying anything (*n* = 532)	65.0 ± 2.1 ^##^	29.7 ± 2.0 ^#^	27.4 ± 1.9	38.7 ± 2.1 ^##^	17.9 ± 1.7 ^##^	11.1 ± 1.4	1.3 ± 0.5 **	8.5 ± 1.2	31.2 ± 2.0 **
	*p* value *	*p* < 0.001 *	*p* = 0.001 *	*p* < 0.001 *	*p* < 0.001 *	*p* < 0.001 *	*p* = 0.219	*p* < 0.001	*p* = 0.076	*p* = 0.003 *
**Strength Training (sessions/week) ^†^**	-	-	-	-	-	-	-	-	-	-
	*p* value	*p* < 0.001 *	*p* < 0.001 *	*p* < 0.001 *	*p* < 0.001 *	*p* < 0.001 *	*p* = 0.009 *	*p* < 0.001 *	*p* = 0.395	*p* < 0.001 *
**Cardio Exercise in Own Time (hours/week) ^†^**	-	-	-	-	-	-	-	-	-	-
	*p* value	*p* = 0.052	*p* = 0.311	*p* = 0.017 *	*p* < 0.001 *	*p* < 0.001 *	*p* = 0.095	*p* = 0.519	*p* = 0.779	*p* = 0.310
**Cardio Exercise within Unit (hours/week) ^†^**	-	-	-	-	-	-	-	-	-	-
	*p* value	*p* = 0.004 *	*p* = 0.841	*p* = 0.553	*p* = 0.014 *	*p* = 0.001 *	*p* = 0.946	*p* = 0.698	*p* = 0.210	*p* = 0.278
**Total Cardio Exercise (hours/week) ^†^**	-	-	-	-	-	-	-	-	-	-
	*p* value	*p* = 0.007 *	*p* = 0.397	*p* = 0.001 *	*p* = 0.685	*p* = 0.347	*p* = 0.080	*p* = 0.123	*p* = 0.647	*p* = 0.003 *

^1^ Logistic regression was used to test for any significant differences in the proportion of users between the subgroups of each categorical variable, and to test for any significant associations in the proportion of users and each continuous variable; DS: dietary supplement; SE: standard error; SOCOMD: special operations command; BMI: body mass index; * p < 0.05 was considered significant; ** significantly different from first subgroup within variable at *p* < 0.05; ^#^ significantly different from first subgroup within variable at *p* < 0.01; ^##^ significantly different from first subgroup within variable at *p* < 0.001; ^†^ continuous variable.

**Table 2:** Odds ratios (ORs) and 95% CIs for the use of each category of DS by demographic, military, and lifestyle characteristics ^1^.

Variable	Subgroup	Any DS	Multivitamin and Mineral	Individual Vitamin or Mineral	Protein or Amino Acid	Combination Product	Herbal Product	Purported Prohormone	Joint Health Product	Other
**Gender**	Male	1.00	1.00	1.00	1.00	1.00	1.00	1.00	1.00	1.00
	Female	2.15 (1.44–3.21) ^##^	1.36 (1.04–1.77) *	2.23 (1.72–2.89) ^##^	0.86 (0.67–1.10)	1.20 (0.93–1.55)	1.61 (1.17–2.23) ^#^	0.52 (0.25–1.07)	1.18 (0.79–1.76)	1.24 (0.97–1.59)
	Prefer not to say	0.52 (0.14–1.96)	0.63 (0.23–1.80)	0.86 (0.31–2.43)	0.39 (0.14–1.03)	0.60 (0.20–1.82)	1.38 (0.40–4.80)	^†^	3.74 (1.32–10.61)	1.08 (0.42–2.80)
	Indeterminate/Intersex/Unspecified	0.38 (0.03–4.21)	0.37 (0.08–1.71)	0.84 (0.22–3.18)	0.29 (0.08–1.09)	0.21 (0.03–1.63)	1.53 (0.33–7.14)	^†^	^†^	0.97 (0.28–3.32)
**Age**	18–22	1.00	1.00	1.00	1.00	1.00	1.00	1.00	1.00	1.00
	23–27	1.65 (1.04–2.61) *	2.31 (1.58–3.37) ^##^	1.58 (1.10–2.25)	1.67 (1.10–2.53) *	1.73 (1.19–2.53) ^#^	1.21 (0.72–2.01)	2.14 (0.87–5.25)	6.53 (1.54–27.72) *	1.75 (1.20–2.55) ^#^
	28–32	1.39 (0.86–2.24)	2.23 (1.51–3.31) ^##^	1.50 (1.04–2.17)	1.44 (0.95–2.19)	1.86 (1.26–2.76) ^#^	1.23 (0.73–2.07)	1.81 (0.72–4.58)	7.91 (1.87–33.55) ^#^	1.81 (1.23–2.66) ^#^
	33–37	1.52 (0.90–2.59)	2.55 (1.68–3.87) ^##^	1.59 (1.08–2.34)	1.81 (1.17–2.81) ^#^	1.83 (1.20–2.79) ^#^	1.29 (0.74–2.22)	1.96 (0.76–5.11)	10.56 (2.48–44.90) ^#^	2.83 (1.87–4.26) ^##^
	38–42	1.07 (0.60–1.89)	2.69 (1.73–4.19) ^##^	1.34 (0.89–2.03)	0.82 (0.52–1.31)	1.22 (0.76–1.93)	1.81 (1.05–3.14)	2.43 (0.92–6.38)	14.18 (3.33–60.43) ^##^	1.98 (1.27–3.09) ^#^
	43–47	0.69 (0.39–1.22)	2.01 (1.27–3.17) ^#^	0.87 (0.57–1.35)	0.58 (0.36–0.92) *	0.70 (0.42–1.67)	1.04 (0.57–1.90)	1.28 (0.44–3.76)	16.42 (3.87–69.64) ^##^	2.18 (1.39–3.41) ^##^
	48–52	0.73 (0.40–1.32)	2.08 (1.30–3.33) ^#^	1.26 (0.82–1.94)	0.37 (0.23–0.62) ^##^	0.49 (0.28–0.85) *	1.80 (1.02–3.18)	1.12 (0.36–3.54)	20.54 (4.84–87.09) ^##^	2.36 (1.49–3.75) ^##^
	53–57	1.27 (0.65–2.51)	1.58 (0.94–2.68)	1.00 (0.61–1.65)	0.54 (0.31–0.93) *	0.63 (0.35–1.16)	0.94 (0.46–1.94)	0.27 (0.03–2.30)	23.45 (5.42–101.40) ^##^	2.94 (1.78–4.85) ^##^
	58–60	1.47 (0.55–3.91)	2.29 (1.03–5.10) *	1.46 (0.68–3.15)	0.24 (0.09–0.67) ^#^	0.21 (0.05–0.93) *	1.08 (0.35–3.35)	3.29 (0.78–13.84)	43.33 (8.96–209.52) ^##^	4.49 (2.06–9.78) ^##^
	Prefer not to say	0.87 (0.19–3.96)	^†^	0.45 (0.10–2.06)	0.76 (0.10–6.00)	0.68 (0.06–7.65)	1.36 (0.29–6.44)	^†^	28.36 (4.29–187.59) ^##^	0.91 (0.10–8.71)
**Command**	Other than SOCOMD	1.00	1.00	1.00	1.00	1.00	1.00	1.00	1.00	1.00
	SOCOMD	1.98 (1.26–3.12)	1.60 (1.19–2.13)	1.64 (1.21–2.23) ^#^	1.64 (1.21–2.23)	1.04 (0.77–1.42)	1.38 (0.94–2.04)	1.58 (0.88–2.84)	0.91 (0.55–1.51)	1.25 (0.93–1.67)
**Rank**	Trainee	1.00	1.00	1.00	1.00	1.00	1.00	1.00	1.00	1.00
	Private—Corporal	0.56 (0.20–1.56)	1.29 (0.64–2.58)	1.06 (0.54–2.10)	0.70 (0.33–1.45)	0.82 (0.43–1.57)	1.35 (0.47–3.85)	2.55 (0.34–18.85)	1.24 (0.29–5.26)	0.81 (0.42–1.57)
	Senior NCO	0.83 (0.28–2.48)	1.40 (0.67–2.91)	1.04 (0.50–2.14)	0.43 (0.20–0.94)	0.63 (0.31–1.26)	1.69 (0.57–5.06)	1.98 (0.25–15.76)	2.56 (0.58–11.24)	1.13 (0.56–2.27)
	Warrant Officer	0.76 (0.26–2.25)	1.21 (0.59–2.50)	0.89 (0.43–1.81)	0.21 (0.10–0.44)	0.29 (0.15–0.59)	1.24 (0.42–3.71)	1.64 (0.21–13.02)	3.15 (0.73–13.58)	0.76 (0.38–1.50)
	Officer Cadet—Junior Commissioned Officer	0.88 (0.30–2.62)	1.75 (0.84–3.63)	0.99 (0.48–2.03)	0.80 (0.37–1.73)	0.85 (0.43–1.70)	1.35 (0.45–4.07)	0.81 (0.09–7.14)	1.78 (0.40–7.93)	0.85 (0.42–1.71)
	Senior Commissioned Officer	0.69 (0.23–2.02)	1.39 (0.68–2.85)	0.65 (0.32–1.33)	0.16 (0.08–0.35)	0.24 (0.12–0.48)	1.17 (0.39–3.45)	0.78 (0.09–6.50)	2.68 (0.62–11.51)	0.75 (0.38–1.48)
	Prefer not to say	0.68 (0.16–3.01)	0.89 (0.29–2.72)	0.79 (0.26–2.39)	0.18 (0.06–0.54)	0.41 (0.13–1.27)	1.70 (0.38–7.56)	^†^	4.74 (0.84–26.76)	1.38 (0.49–3.84)
**Corps Area**	Combat	1.00	1.00	1.00	1.00	1.00	1.00	1.00	1.00	1.00
	Combat Support	1.72 (1.15–2.56) ^#^	1.34 (1.01–1.77)	1.31 (0.98–1.75)	1.09 (0.83–1.45)	1.17 (0.87–1.57)	1.60 (1.09–2.34)	1.52 (0.85–2.73)	1.22 (0.76–1.95)	1.43 (1.08–1.88)
	Combat Service Support	1.35 (1.05–1.73)	1.07 (0.88–1.28)	1.19 (0.98–1.45)	0.88 (0.73–1.05)	1.23 (1.01–1.49)	1.36 (1.03–1.78)	1.05 (0.68–1.62)	1.34 (0.98–1.82)	1.12 (0.93–1.35)
	Officer Training Corps	1.76 (0.16–19.56)	1.04 (0.25–4.36)	3.81 (0.90–16.04)	1.27 (0.30–5.33)	1.38 (0.33–5.81)	2.62 (0.52–13.16)	^†^	^†^	0.60 (0.12–3.01)
**BMI Range**	<25	1.00	1.00	1.00	1.00	1.00	1.00	1.00	1.00	1.00
	25–30	1.03 (0.72–1.48)	1.31 (1.07–1.59)	0.97 (0.80–1.17)	1.11 (0.92–1.34)	1.28 (1.01–1.62) *	1.03 (0.78–1.36)	1.82 (1.11–2.99)	1.77 (1.23–2.54) ^#^	1.31 (1.08–1.60)
	>30	0.83 (0.42–1.64)	1.13 (0.85–1.50)	0.87 (0.65–1.16)	0.80 (0.61–1.05)	1.57 (1.11–2.23) *	1.03 (0.69–1.53)	1.67 (0.85–3.27)	1.08 (0.65–1.81)	1.28 (0.97–1.70)
**BMI (kg/m^2^) ^††^**	-	1.05 (0.98–1.12)	1.02 (1.00–1.05)	0.98 (0.96–1.01)	0.99 (0.97–1.02)	1.05 (1.02–1.08)	1.02 (0.98–1.06)	1.09 (1.03–1.17)	1.06 (1.02–1.10)	1.04 (1.01–1.06)
**Body Weight Actions**	Trying to lose weight	1.00	1.00	1.00	1.00	1.00	1.00	1.00	1.00	1.00
	Trying to gain weight	1.62 (0.99–2.64)	0.99 (0.72–1.35)	1.06 (0.77–1.45)	2.14 (1.42–3.24) ^##^	0.74 (0.53–1.04)	0.91 (0.61–1.36)	1.60 (0.82–3.12)	0.64 (0.37–1.08)	0.80 (0.58–1.09)
	Trying to maintain weight	1.09 (0.79–1.49)	1.13 (0.90–1.43)	1.46 (1.15–1.85) ^#^	1.15 (0.89–1.49)	0.89 (0.62–1.15)	1.09 (0.80–1.48)	2.17 (1.30–3.64)	1.24 (0.88–1.75)	1.16 (0.92–1.46)
	Not trying anything	0.53 (0.40–0.71) ^##^	0.67 (0.52–0.85) ^#^	0.85 (0.66–1.09)	0.50 (0.38–0.64) ^##^	0.38 (0.28–0.51) ^##^	0.75 (0.54–1.05)	0.42 (0.18–0.98)	0.84 (0.58–1.24)	0.74 (0.58–0.94) *
**Strength Training (sessions/week) ^††^**	-	1.30 (1.23–1.34)	1.09 (1.05–1.13)	1.11 (1.08–1.16)	1.52 (1.46–1.59)	1.28 (1.23–1.34)	1.06 (1.01–1.11)	1.20 (1.12–1.28)	0.98 (0.93–1.03)	1.15 (1.11–1.19)
**Cardio Exercise in Own Time (hours/week) ^††^**	-	1.03 (1.00–1.07)	1.01 (0.99–1.04)	1.05 (1.03–1.08)	1.04 (1.02–1.07)	1.05 (1.02–1.07)	1.03 (1.01–1.06)	1.04 (1.00–1.08)	1.01 (0.97–1.04)	1.04 (1.02–1.06)
**Cardio Exercise within Unit (hours/week) ^††^**	-	0.94 (0.90–0.98)	1.00 (0.97–1.04)	1.01 (0.98–1.04)	1.04 (1.01–1.08)	1.05 (1.02–1.09)	1.00 (0.96–1.05)	1.01 (0.95–1.09)	0.96 (0.91–1.02)	1.02 (0.99–1.05)
**Total Cardio Exercise (hours/week) ^††^**	-	^†^	1.01 (0.99–1.02)	1.03 (1.01–1.05)	1.04 (1.02–1.06)	1.04 (1.02–1.06)	1.02 (1.00–1.04)	1.03 (0.99–1.06)	0.99 (0.97–1.02)	1.03 (1.01–1.04)

^1^ Logistic regression was used to test for any significant differences in the proportion of users between the subgroups of each categorical variable, and to test for any significant associations in the proportion of users and each continuous variable; ODs: odds ratios; CI: confidence intervals; DS: dietary supplement; SOCOMD: special operations command; BMI: body mass index; * significantly different from first subgroup within each variable at *p* < 0.05; ^#^ significantly different from first subgroup within each variable at *p* < 0.01; ^##^ significantly different from first subgroup within each variable at *p* < 0.001; ^†^ insufficient data; ^††^ continuous variable.

**Table 3:** Prevalence and predictors of the use of each category of nutritional supplement (NS) ≥ 1 times/week by demographic, military, and lifestyle characteristics ^1^.

Variable	Subgroup	Any NS (% ± SE)	Sports Drink (% ± SE)	Sports Bar or Gel (% ± SE)	Meal Replacement Beverage (% ± SE)
**Group**	All (*n* = 2162)	21.5 ± 0.9	19.5 ± 0.9	4.3 ± 0.4	0.3 ± 0.1
**Gender**	Male (*n* = 1833)	21.7 ± 1.0	19.6 ± 0.9	4.1 ± 0.5	0.2 ± 0.1
	Female (*n* = 296)	20.9 ± 2.4	18.9 ± 2.3	5.1 ± 1.3	0.7 ± 0.5
	Prefer not to say (*n* = 18)	22.2 ± 9.8	22.2 ± 9.8	5.6 ± 5.4	0.0 ± 0.0
	Indeterminate/Intersex/Unspecified (*n* = 11)	9.1 ± 8.7	9.1 ± 8.7	0.0 ± 0.0	0.0 ± 0.0
	*p* value *	*p* = 0.904	*p* = 0.927	*p* = 0.449	^†^
**Age**	18–22 (*n* = 210)	20.0 ± 2.8	19.0 ± 2.7	1.4 ± 0.8	^†^
	23–27 (*n* = 457)	29.3 ± 2.1 **	28.2 ± 2.1 **	3.5 ± 0.9	^†^
	28–32 (*n* = 396)	19.4 ± 2.0	17.9 ± 1.9	4.0 ± 1.0	^†^
	33–37 (*n* = 293)	23.2 ± 2.5	19.1 ± 2.3	5.8 ± 1.4	^†^
	38–42 (*n* = 225)	20.4 ± 2.7	17.8 ± 2.5	5.3 ± 1.5	^†^
	43–47 (*n* = 220)	16.4 ± 2.5	14.1 ± 2.3	5.0 ± 1.5	^†^
	48–52 (*n* = 188)	19.7 ± 2.9	17.0 ± 2.7	4.3 ± 1.5	^†^
	53–57 (*n* = 125)	15.2 ± 3.2	13.6 ± 3.1	4.8 ± 1.9	^†^
	58–60 (*n* = 34)	8.8 ± 4.9	8.8 ± 4.9	2.9 ± 2.9	^†^
	Prefer not to say (*n* = 14)	21.4 ± 11.0	14.3 ± 9.4	14.3 ± 9.4	^†^
	*p* value *	*p* < 0.001 *	*p* < 0.001 *	*p* = 0.352	^†^
**Command**	SOCOMD (*n* = 202)	19.8 ± 2.8	19.7 ± 0.9 *	5.4 ± 1.6	^†^
	Other than SOCOMD (*n* = 1960)	21.7 ± 0.9	17.3 ± 2.7	4.1 ± 0.4	^†^
	*p* value *	*p* = 0.536	*p* = 0.419	*p* = 0.380	^†^
**Rank**	Trainee (*n* = 38)	10.5 ± 5.0	10.5 ± 5.0	0.0 ± 0.0	^†^
	Private—Corporal (*n* = 1025)	24.4 ± 1.3	22.6 ± 1.3	3.7 ± 0.6	^†^
	Senior NCO (*n* = 217)	20.3 ± 2.7	17.5 ± 2.6	4.6 ± 1.4	^†^
	Warrant Officer (*n* = 282)	19.1 ± 2.3	17.0 ± 2.2	3.9 ± 1.2	^†^
	Officer Cadet—Junior Commissioned Officer (*n* = 233)	20.2 ± 2.6	19.3 ± 2.6	3.9 ± 1.3	^†^
	Senior Commissioned Officer (*n* = 343)	17.4 ± 2.1	14.1 ± 1.9	6.2 ± 1.3	^†^
	Prefer not to say (*n* = 24)	25.0 ± 8.8	25.0 ± 8.8	8.3 ± 5.6	^†^
	*p* value *	*p* = 0.681	*p* = 0.681	*p* = 0.278	^†^
**Corps Area**	Combat (*n* = 913)	20.9 ± 1.3	19.6 ± 1.3	3.1 ± 0.6	^†^
	Combat Support (*n* = 261)	21.8 ± 2.6	19.9 ± 2.5	4.6 ± 1.3	^†^
	Combat Service Support (*n* = 980)	21.9 ± 1.3	19.2 ± 1.3	5.2 ± 0.7	^†^
	Officer Training Corps (*n* = 8)	25.0 ± 15.3	25.0 ± 15.3	12.5 ± 11.7	^†^
	*p* value *	*p* = 0.947	*p* = 0.969	*p* = 0.091	^†^
**BMI**	<25 (*n* = 702)	22.1 ± 1.6	19.2 ± 1.5	5.7 ± 0.9	^†^
	25–30 (*n* = 1116)	21.4 ± 1.2	19.7 ± 1.2	3.8 ± 0.6	^†^
	>30 (*n* = 292)	21.8 ± 2.4	20.4 ± 2.4	3.1 ± 1.0	^†^
	*p* value *	*p* = 0.944	*p* = 0.910	*p* = 0.083	^†^
**BMI (kg/m^2^)** **^††^**	-	-	-	-	-
	*p* value	*p* = 0.419	*p* = 0.780	*p* = 0.040	^†^
**Body Weight Actions**	Trying to lose weight (*n* = 810)	22.1 ± 1.5	19.6 ± 1.4	4.2 ± 0.7	^†^
	Trying to gain weight (*n* = 276)	23.6 ± 2.6	22.5 ± 2.5	2.9 ± 1.0	^†^
	Trying to maintain weight (*n* = 544)	23.7 ± 1.8	21.3 ± 1.8	5.9 ± 1.0	^†^
	Not trying anything (*n* = 532)	17.3 ± 1.6	15.8 ± 1.6	3.4 ± 0.8	^†^
	*p* value *	*p* = 0.077	*p* = 0.154	*p* = 0.130	^†^
**Strength Training (sessions/week) ^††^**	-	-	-	-	-
	*p* value	*p* = 0.348	*p* = 0.207	*p* < 0.457	^†^
**Cardio Exercise in Own Time (hours/week) ^††^**	-	-	-	-	-
	*p* value	*p* < 0.001 *	*p* < 0.001 *	*p* < 0.001 *	^†^
**Cardio Exercise within Unit (hours/week) ^††^**	-	-	-	-	-
	*p* value	*p* < 0.001 *	*p* < 0.001 *	*p* = 0.017 *	^†^
**Total Cardio Exercise (hours/week) ^††^**	-	-	-	-	-
	*p* value	*p* < 0.001 *	*p* < 0.001 *	*p* < 0.001 *	^†^

^1^ Logistic regression was used to test for any significant differences in the proportion of users between the subgroups of each categorical variable, and to test for any significant associations in the proportion of users and each continuous variable NS: nutritional supplement; SE: standard error; SOCOMD: special operations command; BMI: body mass index; * p < 0.05 considered significant; ** significantly different from first subgroup within each variable at *p* < 0.05; ^†^ insufficient data; ^††^ continuous variable.

**Table 4:** ORs and 95% CIs for the use of each category of NS by demographic, military, and lifestyle characteristics ^1^.

Variable	Subgroup	Any NS	Sports Drink	Sports Bar or Gel	Meal Replacement Beverage
**Gender**	Male (*n* = 1833)	1.00	1.00	1.00	^†^
	Female (*n* = 296)	0.96 (0.71–1.23)	0.96 (0.70–1.31)	1.25 (0.71–2.21)	^†^
	Prefer not to say (*n* = 18)	1.03 (0.34–3.16)	1.17 (0.38–3.57)	1.38 (0.18–10.50)	^†^
	Indeterminate/Intersex/Unspecified (*n* = 11)	0.36 (0.05–2.83)	0.41 (0.05–3.21)	^†^	^†^
**Age**	18–22 (*n* = 210)	1.00	1.00	1.00	^†^
	23–27 (*n* = 457)	1.67 (1.12–2.48) *	1.67 (1.12–2.51) *	2.50 (0.72–8.69)	^†^
	28–32 (*n* = 396)	0.97 (0.64–1.49)	0.94 (0.61–1.44)	2.91 (0.84–10.09)	^†^
	33–37 (*n* = 293)	1.27 (0.82–1.96)	1.05 (0.67–1.65)	4.25 (1.23–14.69)	^†^
	38–42 (*n* = 225)	1.04 (0.65–1.66)	0.92 (0.57–1.51)	3.89 (1.08–13.98)	^†^
	43–47 (*n* = 220)	0.78 (0.47–1.28)	0.70 (0.41–1.16)	3.63 (1.00–13.21)	^†^
	48–52 (*n* = 188)	0.95 (0.58–1.57)	0.85 (0.51–1.42)	3.07 (0.80–11.73)	^†^
	53–57 (*n* = 125)	0.68 (0.37–1.24)	0.64 (0.34–1.19)	3.48 (0.85–14.17)	^†^
	58–60 (*n* = 34)	0.38 (0.11–1.32)	0.41 (0.12–1.41)	2.09 (0.21–20.71)	^†^
	Prefer not to say (*n* = 14)	1.08 (0.29–4.07)	0.70 (0.15–3.27)	11.50 (1.75–75.48)	^†^
**Command**	Other than SOCOMD (*n* = 1960)	1.00	1.00	1.00	^†^
	SOCOMD (*n* = 202)	0.89 (0.62–1.28)	0.86 (0.58–1.25)	1.37 (0.70–2.56)	^†^
**Rank**	Trainee (*n* = 38)	1.00	1.00	1.00	^†^
	Private—Corporal (*n* = 1025)	2.74 (0.96–7.80)	2.49 (0.87–7.08)	^†^	^†^
	Senior NCO (*n* = 217)	2.16 (0.73–6.41)	1.80 (0.61–5.39)	^†^	^†^
	Warrant Officer (*n* = 282)	2.01 (0.69–5.91)	1.74 (0.59–5.14)	^†^	^†^
	Officer Cadet—Junior Commissioned Officer (*n* = 233)	2.15 (0.73–6.35)	2.04 (0.69–6.03)	^†^	^†^
	Senior Commissioned Officer (*n* = 343)	1.78 (0.61–5.22)	1.40 (0.48–4.12)	^†^	^†^
	Prefer not to say (*n* = 24)	2.83 (0.71–11.36)	2.83 (0.71–11.36)	^†^	^†^
**Corps Area**	Combat (*n* = 913)	1.00	1.00	1.00	^†^
	Combat Support (*n* = 261)	1.06 (0.76–1.48)	1.02 (0.72–1.44)	0.69 (0.31–1.50)	^†^
	Combat Service Support (*n* = 980)	1.06 (0.85–1.32)	0.97 (0.78–1.22)	1.43 (0.87–2.34)	^†^
	Officer Training Corps (*n* = 8)	1.26 (0.25–6.29)	1.37 (0.27–6.83)	0.80 (0.45–1.43)	^†^
**BMI**	<25 (*n* = 702)	1.00	1.00	1.00	^†^
	25–30 (*n* = 1116)	0.96 (0.77–1.21)	1.03 (0.81–1.31)	0.65 (0.42–1.01)	^†^
	>30 (*n* = 292)	0.98 (0.71–1.37)	1.08 (0.77–1.52)	0.53 (0.26–1.11)	^†^
**BMI (kg/m^2^) ^††^**	-	0.99 (0.96–1.02)	1.00 (0.96–1.03)	0.93 (0.87–1.00)	^†^
**Body Weight Actions**	Trying to lose weight (*n* = 810)	1.00	1.00	1.00	^†^
	Trying to gain weight (*n* = 276)	1.09 (0.79–1.50)	1.19 (0.85–1.65)	0.68 (0.31–1.50)	^†^
	Trying to maintain weight (*n* = 544)	1.10 (0.85–1.42)	1.11 (0.85–1.45)	1.43 (0.87–2.34)	^†^
	Not trying anything (*n* = 532)	0.74 (0.56–0.98)	0.77 (0.57–1.03)	0.80 (0.45–1.43)	^†^
**Strength Training (sessions/week) ^††^**	-	1.08 (1.04–1.12)	1.09 (1.05–1.13)	1.03 (0.96–1.11)	^†^
**Cardio Exercise in Own Time (hours/week) ^††^**	-	1.07 (1.04–1.09)	1.06 (1.03–1.08)	1.13 (1.10–1.17)	^†^
**Cardio Exercise within Unit (hours/week) ^††^**	-	1.07 (1.04–1.11)	1.08 (1.04–1.11)	1.07 (1.01–1.13)	^†^
**Total Cardio Exercise (hours/week) ^††^**	-	1.06 (1.04–1.08)	1.05 (1.03–1.07)	1.09 (1.06–1.12)	^†^

^1^ Logistic regression was used to test for any significant differences in the proportion of users between the subgroups of each categorical variable, and to test for any significant associations in the proportion of users and each continuous variable; ORs: odds ratios; CI: confidence intervals; NS: nutritional supplement; SE: standard error; SOCOMD: special operations command; BMI: body mass index; * significantly different from first subgroup within each variable at *p* < 0.05; ^†^ insufficient data; ^††^ continuous variable.

**Table 5:** Characteristics of regular DS users reporting ≥1 side effects.

Variable	Subgroup	% ± SE
**Group**	All (*n* = 267)	15.9 ± 0.1
**Gender**	Male (*n* = 220)	82.4 ± 2.3
	Female (*n* = 45)	16.9 ± 2.3
	Prefer not to say (*n* = 1)	0.4 ± 0.4
	Indeterminate/Intersex/Unspecified (*n* = 1)	0.4 ± 0.4
**Age**	18–22 (*n* = 34)	12.7 ± 2.0
	23–27 (*n* = 76)	28.5 ± 2.8
	28–32 (*n* = 67)	25.1 ± 2.7
	33–37 (*n* = 46)	17.2 ± 2.3
	38–42 (*n* = 20)	7.5 ± 1.6
	43–47 (*n* = 9)	3.4 ± 1.1
	48–52 (*n* = 10)	3.8 ± 1.2
	53–57 (*n* = 5)	1.9 ± 0.8
	58–60 (*n* = 0)	0.0 ± 0.0
**Corps Area**	Combat (*n* = 109)	40.8 ± 3.0
	Combat Support (*n* = 34)	12.7 ± 2.0
	Combat Service Support (*n* = 121)	45.3 ± 3.1
	Officer Training Corps (*n* = 3)	1.1 ± 0.7
**BMI Range**	<25 (*n* = 76)	28.5 ± 2.8
	25–30 (*n* = 145)	54.3 ± 3.1
	>30 (*n* = 37)	13.9 ± 2.1
**Body Weight Actions**	Trying to lose weight	42.7 ± 3.0
	Trying to gain weight	15.4 ± 2.2
	Trying to maintain weight	30.0 ± 2.8
	Not trying anything	12.0 ± 2.0

SE: standard error; BMI: body mass index.

**Table 6:** Significant predictors of the number of DSs and NSs used.

Supplement	Variable	Subgroup	OR (95% CI)	*p* Value
**DSs**	**Gender**			<0.001 *
		Male	1.00	-
		Female	1.43 (1.23–1.66)	<0.001 *
	**Age**			<0.001 *
		18–22	1.00	-
		23–27	1.58 (1.30–1.91)	<0.001 *
		28–32	1.56 (1.28–1.90)	<0.001 *
		33–37	1.73 (1.40–2.14)	<0.001 *
		38–42	1.51 (1.20–1.90)	0.001 *
		43–47	1.23 (0.97–1.55)	0.092
		48–52	1.31 (1.02–1.67)	0.033 *
		53–57	1.13 (0.86–1.48)	0.399
		58–60	1.16 (0.74–1.81)	0.524
		Prefer not to say	0.21 (0.02–2.05)	0.182
	**BMI Range**			0.005 *
		<25	1.00	-
		25–30	1.21 (1.08–1.37)	0.001 *
		>30	1.19 (1.00–1.42)	0.045 *
	**Body Weight Actions**			<0.001 *
		Trying to lose weight	1.00	-
		Trying to gain weight	1.08 (0.91–1.29)	0.016 *
		Trying to maintain weight	1.17 (1.03–1.34)	0.377
		Not trying anything	0.73 (0.64–0.84)	<0.001 *
	Strength Training (sessions/week) **		1.16 (1.14–1.18)	<0.001 *
**NSs**	**Age group**			<0.001 *
		18–22	1.00	-
		23–27	1.61 (1.20–2.17)	0.002 *
		28–32	1.11 (0.81–1.52)	0.523
		33–37	1.28 (0.92–1.78)	0.140
		38–42	1.11 (0.78–1.59)	0.559
		43–47	0.92 (0.63–1.33)	0.642
		48–52	0.96 (0.66–1.40)	0.829
		53–57	0.83 (0.53–1.28)	0.389
		58–60	0.47 (0.20–1.14)	0.095
		Prefer not to say	1.33 (0.52–3.38)	0.546
	Cardio Exercise in Own Time (hours/week) **		1.08 (1.06–1.10)	<0.001 *

DSs: dietary supplements; NSs: nutritional supplements; OR: odds ratios; CI: confidence intervals; BMI: body mass index; * *p* < 0.05 considered significant; ** continuous variable.

**Table 7:** Pearson correlation coefficients between continuous variables and number of DS used.

Variable	Number of DSs Used	*p* Value	Number of NSs Used	*p* Value
BMI (kg/m^2^)	0.051	0.019 *	−0.023 *	0.288
Cardio exercise at work (hours/week)	0.075	<0.001 *	0.110	<0.001 *
Cardio in own time (hours/week)	0.098	<0.001 *	0.191	<0.001 *
Cardio total (hours/week)	0.111	<0.001 *	0.197	<0.001 *
Strength conditioning sessions within the unit (sessions/week)	0.050	0.020 *	0.065	0.003 *
Strength conditioning sessions in own time (sessions/week)	0.431	<0.001 *	0.068	0.002 *
Strength conditioning sessions in total (sessions/week)	0.380	<0.001 *	0.094	<0.001 *

DSs: dietary supplements; NSs: nutritional supplements; BMI: body mass index; * p < 0.05 considered significant.
